# Relationship between oxidative balance score and post-stroke depression: insights from the NHANES 2005–2018 cross-sectional study

**DOI:** 10.3389/fneur.2024.1440761

**Published:** 2024-11-25

**Authors:** Hai-Jun Li, Bo Gao, Li-Ming Yan, Yi-Dong Xue, Tao Wang

**Affiliations:** ^1^Department of Neurology, The Affiliated Hospital of Yan'an University, Yan'an, Shaanxi, China; ^2^Department of Gynecology, The Affiliated Hospital of Yan'an University, Yan'an, Shaanxi, China

**Keywords:** oxidative balance score, post-stroke depression, PHQ-9, NHANES, stroke

## Abstract

**Introduction:**

The Oxidative Balance Score (OBS) represents an integrated measure of oxidative-reductive homeostasis. Despite the established role of oxidative stress in the development of post-stroke depression (PSD), the association between OBS and PSD in the general population remains unclear. This study aims to explore this relationship using data from the National Health and Nutrition Examination Survey (NHANES) spanning 2005–2018.

**Methods:**

The OBS was calculated using validated methods that incorporate dietary and lifestyle factors, whereas PSD status was determined using self-reported data and the Patient Health Questionnaire-9 (PHQ-9) scores. Multivariable logistic regression was employed to evaluate the associations of total OBS, dietary OBS, and lifestyle OBS with PSD prevalence, adjusting for potential confounders. Dose–response relationships were further assessed using restricted cubic splines (RCS).

**Results:**

Among the 26,668 participants included in the analysis, 201 were diagnosed with PSD. After adjusting for confounding variables, higher total OBS, dietary OBS, and lifestyle OBS were each significantly associated with reduced odds of PSD. The adjusted odds ratios (OR) and 95% confidence intervals (CI) for the highest versus lowest quartiles were 0.31 (95% CI: 0.15–0.67) for total OBS, 0.45 (0.27–0.73) for dietary OBS, and 0.28 (0.13–0.60) for lifestyle OBS. RCS analyses indicated a linear dose–response relationship for all three OBS categories with PSD risk. In sex-stratified analyses, significant inverse associations were observed between all OBS categories and PSD in females, whereas only lifestyle OBS was significantly associated with PSD in males.

**Conclusion:**

Higher OBS was associated with reduced odds of PSD, with a particularly pronounced effect in females. These findings suggest that adherence to an antioxidant-rich diet and lifestyle may mitigate PSD risk.

## 1 Introduction

Stroke, which includes both ischemic and hemorrhagic types, is the second leading cause of disability and mortality globally, imposing a significant health burden across both low- and high-income countries (1, 2). However, the complications following a stroke often present even greater challenges than the stroke itself. Common complications among stroke survivors include depression, anxiety, fatigue, apathy, insomnia, mania, and cognitive impairment (3). Among these, post-stroke depression (PSD) is one of the most prevalent, affecting approximately one-third of stroke survivors, with its incidence increasing over time (4). Patients with PSD typically experience persistent low mood and a notable loss of interest in previously enjoyable activities, alongside symptoms such as psychomotor agitation, feelings of worthlessness, sleep disturbances, weight fluctuations, and suicidal ideation, often persisting for more than 2 weeks (5, 6). Despite its significant impact on post-stroke functional recovery and quality of life (7), PSD remains frequently under-recognized and undertreated, and its underlying pathogenesis is still a subject of debate.

Oxidative stress, characterized by an imbalance between antioxidant defenses and pro-oxidant mechanisms, results in oxidative damage to neural tissue through the overproduction of reactive oxygen species (ROS). Elevated ROS levels contribute to cellular injury, lipid peroxidation, and mitochondrial dysfunction, which collectively exacerbate both stroke and depression (8, 9). In the context of stroke, heightened oxidative stress has been implicated in the pathophysiology of PSD, playing a significant role in its development by damaging neural cells and disrupting neuroplasticity (10, 11). Given the significant role of oxidative stress in both stroke and depression, understanding how to effectively assess and manage oxidative stress is crucial. However, due to the complex and dynamic interactions between pro-oxidants and antioxidants, evaluating oxidative homeostasis through a single oxidative stress-related marker is insufficient.

To address this complexity, the Oxidative Balance Score (OBS) was developed as a composite metric that quantifies exposure to both pro-oxidant and antioxidant factors in diet and lifestyle, providing a more comprehensive representation of an individual’s overall oxidative stress burden (12). The OBS comprises 14 dietary antioxidants, 2 dietary pro-oxidants, and 4 lifestyle factors (physical activity, body mass index [BMI], alcohol consumption, and smoking), enabling a thorough assessment of oxidative stress. A higher OBS indicates a reduced oxidative stress burden, characterized by a predominance of antioxidants over pro-oxidants (13, 14).

The OBS has been widely used to reflect oxidative stress exposure and inflammation levels in studies involving American adults, and previous research has demonstrated that OBS is inversely associated with several diseases, including cardiovascular disease, hypertension, chronic kidney disease, diabetes, metabolic syndrome, and stroke (15–17). Despite the growing evidence linking oxidative stress with various health outcomes, no studies have systematically evaluated the relationship between OBS and PSD. Therefore, this study aims to explore the association between OBS and PSD using data from the National Health and Nutrition Examination Survey (NHANES) 2005–2018.

## 2 Methods and materials

### 2.1 Study population

In the United States, NHANES is an ongoing, cross-sectional, nationally representative survey. As a primary initiative of the National Center for Health Statistics (NCHS), NHANES is approved and sponsored by the Centers for Disease Control and Prevention (CDC) to assess the health and nutritional status of the civilian U.S. population. The survey collects data biennially using a complex multistage probability sampling design, including in-home interviews and physical examinations at Mobile Examination Centers (MEC), where blood and urine samples are collected. NHANES is reviewed and approved by the NCHS Research Ethics Review Board (ERB), and informed consent is obtained from all participants [ERB approval protocols for each cycle can be found here].^1^ NHANES data are publicly accessible; for more information, visit the NHANES official website at.^2^

**Figure 1:**
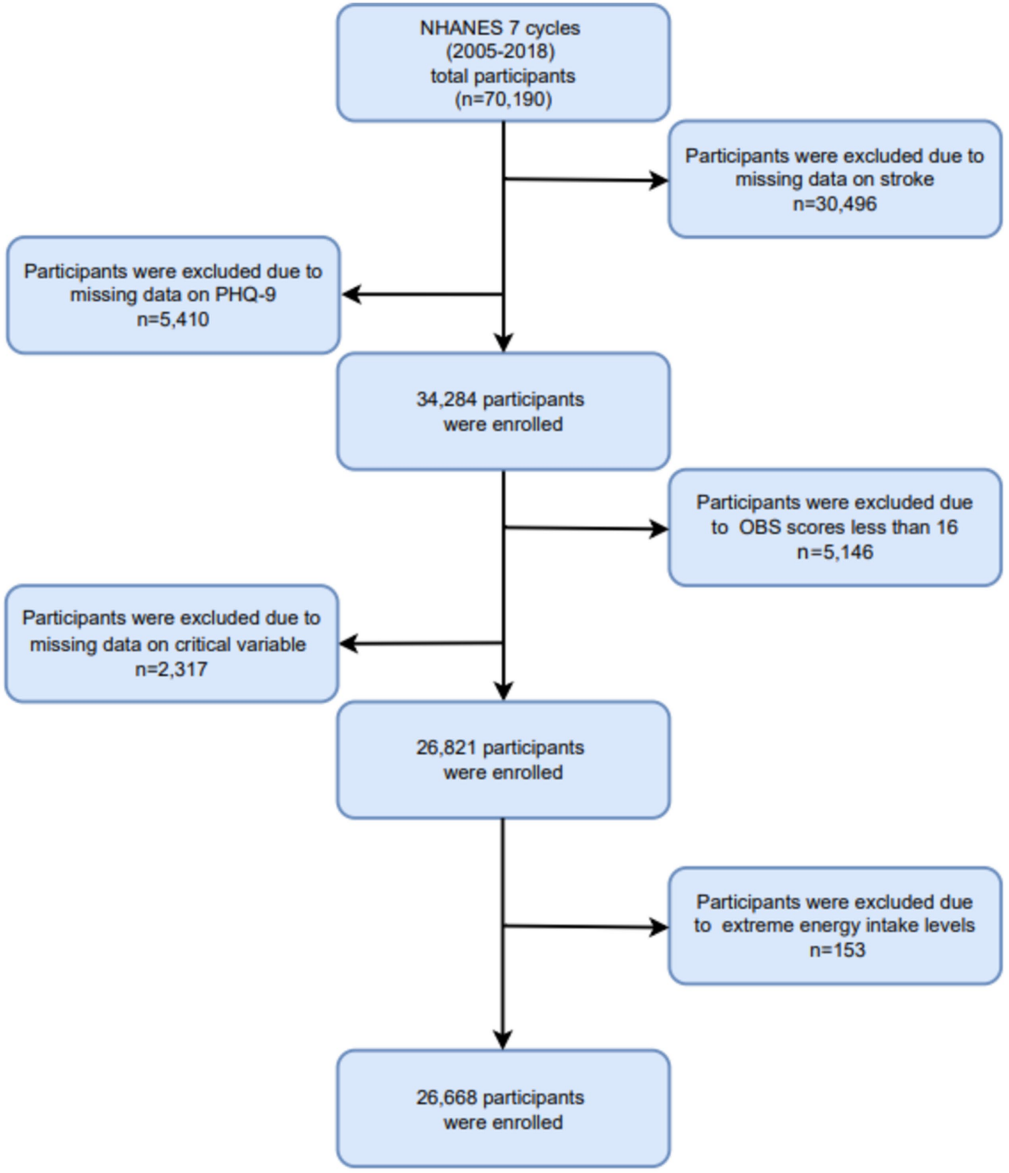
The flowchart of the sample design.

In this study, we analyzed data from 7 cycles of the NHANES database spanning from 2005 to 2018, involving a cohort of 70,190 participants. Participants were excluded if they were missing critical information, including: (1) absence of stroke data (*n* = 30,496); (2) missing PHQ-9 data (*n* = 5,410); (3) incomplete or missing OBS scores, or OBS values below 16 (*n* = 5,146); (4) incomplete essential covariate data (*n* = 2,317); and (5) extreme energy intake values falling outside plausible ranges (men: >8,000 or < 500 kcal/day; women: >5,000 or < 500 kcal/day) (*n* = 153). Ultimately, a total of 26,668 participants were included in the final analysis. The participant selection process is depicted in Figure 1.

### 2.2 Assessment of PSD

Stroke status was ascertained based on participants’ responses to the NHANES survey question: “Has a doctor or other health professional ever informed you that you had a stroke?” (18). Depressive symptoms were assessed using the Patient Health Questionnaire-9 (PHQ-9) (19). Which quantifies the frequency of depressive symptoms experienced over the previous 2 weeks (20). Each of the nine items was scored from 0 to 3, corresponding to the response options: “not at all,” “several days,” “more than half the days,” and “nearly every day,” yielding a total score ranging from 0 to 27. In accordance with previous studies, depression was defined as a PHQ-9 score of ≥10 (21). Participants with both a history of stroke and a PHQ-9 score of 10 or higher were classified as having PSD (22).

### 2.3 Assessment of OBS

The methodologies for constructing and calculating the OBS have been thoroughly detailed in previous literature (23). The OBS is formulated using 16 dietary nutrients along with 4 key lifestyle components. These 20 components were classified into pro-oxidants—total fat, iron, alcohol intake, body mass index (BMI), and cotinine—and antioxidants, including dietary fiber, *β*-carotene, vitamins (B2, niacin, B6, total folate, B12, C, E), minerals (calcium, magnesium, zinc, copper, selenium), and physical activity. Dietary nutrient and alcohol intake were assessed based on the average of two 24-h dietary recalls. In accordance with previous studies, dietary supplements and medication intake were excluded from the analysis (23). Physical activity was quantified using metabolic equivalents (METs), derived from questionnaire data. Serum cotinine was utilized as a biomarker for both active and passive smoking, providing an accurate reflection of individual smoking levels. The overall OBS is calculated as the sum of scores for each component. Antioxidant components were assigned scores of 0, 1, and 2 from the lowest to highest tertiles, whereas pro-oxidants were scored inversely. Table 1 provides a summary of the OBS components and their scoring criteria.

**Table 1:** Component and sex-specific scores assigned to OBS.

	Property	Male			Female		
		0	1	2	0	1	2
Dietary OBS components							
Dietary fiber (g/day)	A	<12.56	12.56–19.67	>19.67	<10.10	10.10–16.30	>16.30
Carotene (RE/day)	A	<98.62	98.62–305.85	>305.85	<98.06	98.06–383.92	>383.92
Riboflavin (mg/day)	A	<1.79	1.79–2.69	>2.69	<1.34	1.34–2.02	>2.02
Niacin (mg/day)	A	<20.64	20.64–29.75	>29.75	<14.51	14.51–21.85	>21.85
Vitamin B6 (mg/day)	A	<1.59	1.59–2.40	>2.40	<1.13	1.13–1.77	>1.77
Total folate (mcg/day)	A	<316.00	316.00–491.94	>491.94	<251.50	251.50–388.5	>388.5
Vitamin B12 (mcg/day)	A	<3.36	3.36–6.20	>6.20	<2.22	2.22–4.21	>4.21
Vitamin C (mg/day)	A	<42.40	42.40–113.20	>113.20	<38.01	38.01–98.40	>98.40
Vitamin E (ATE mg/day)	A	<5.82	5.82–9.41	>9.41	<4.53	4.53–7.51	>7.51
Calcium (mg/day)	A	<645.50	645.50–1071.50	>1071.50	<499.23	499.23–848.78	>848.78
Magnesium (mg/day)	A	<257.00	257.00–361.05	>361.05	<187.00	187.00–283.21	>283.21
Zinc (mg/day)	A	<9.75	9.75–15.10	>15.10	<6.73	6.73–10.74	>10.74
Copper (mg/day)	A	<1.12	1.12–1.57	>1.57	<0.85	0.85–1.28	>1.28
Selenium (mcg/day)	A	<94.94	94.94–141.75	>141.75	<67.75	67.75–99.45	>99.45
Total fat (g/day)	A	>107.42	69.83–107.42	<69.83	>75.78	50.98–75.78	<50.98
Iron (mg/day)	A	>19.16	12.88–19.16	<12.88	>14.32	9.65–14.32	<9.65
Lifestyle OBS components							
Physical activity (MET-minute/week)	A	<417.90	417.90–1135.40	>1135.40	<270.67	270.67–843.27	>843.27
Alcohol (drinks/day)	P	>3 drinks/day	2–3 drinks/day	<=2 drinks/day	>2 drinks/day	1–2 drinks/day	<=1 drinks/day
BMI (kg/m^2^)	P	>29.17	25.55–29.17	<=25.55	>28.63	23.75–28.63	<=23.75
Cotinine (ng/mL)	P	>1.13	0.04–1.13	<0.04	>0.17	0.04–0.17	<0.04

OBS, oxidation balance score; A, antioxidant; P, pro-oxidant.

### 2.4 Covariates assessments

Several key potential covariates were selected based on previous research, including age, gender (male or female), race, education level, marital status, poverty index ratio (PIR), hyperlipidemia, hypertension, and total energy intake per day. Race categories were defined as non-Hispanic white, non-Hispanic black, Mexican American, other Hispanic, or other races. Education level was classified into three groups: less than high school, high school, and more than high school. The poverty index ratio was categorized as 0 to 1.5, 1.5 to 3.5, and > 3.5; hyperlipidemia and hypertension were classified as binary variables (yes or no).

### 2.5 Statistical analyses

Given the complex design of NHANES, and to ensure our sample was representative of the entire US population, we applied sample weights according to the NHANES analysis guidelines. In the baseline analysis, the study population was characterized using continuous variables (mean ± standard error) and categorical variables (percentages). Continuous variables were analyzed with Student’s *t*-test, whereas categorical variables were analyzed using the chi-square test. Multivariable weighted logistic regression was employed to explore the associations between total OBS, dietary OBS, lifestyle OBS, and PSD. Three models were constructed: Model 1 was an unadjusted model; Model 2 included partial adjustments for age, gender, race, marital status, and education level; Model 3 included further adjustments for income level, energy intake, hyperlipidemia, and hypertension based on Model 2 (22). To investigate potential dose–response relationships between OBS and PSD, restricted cubic spline (RCS) analyses were employed. Finally, subgroup analyses stratified by sex were conducted.

All analyses were conducted using the “nhanesR” package in R software (version 4.2.2) and weighted logistic regression analyses were performed using the “survey” package. In all analyses, a *p* < 0.05 was considered statistically significant.

## 3 Results

### 3.1 Baseline characteristics of NHANES participants (2005–2018)

Table 2 presents the baseline characteristics of NHANES participants. After excluding participants with missing key values, 201 participants were identified as having PSD from 2005 to 2018. The weighted mean age of the PSD group was 57.75 years, which was significantly higher than the mean age of 47.41 years in the non-PSD group. Compared to participants without PSD, those with PSD had reduced educational attainment, diminished daily energy intake, and an increased prevalence of hypertension and hyperlipidemia, as well as elevated total OBS, dietary OBS, and lifestyle OBS scores.

**Table 2:** Characteristics of the study population from NHANES 2005–2018.

	Overall (*N* = 26,668)	Non-PSD (26,467)	PSD (*N* = 201)	*p*
Age, mean (SD)	47.46 (16.88)	47.41 (16.88)	57.75 (12.31)	<0.001
Sex, (*n*/%)				0.081
male	12,855 (48.2)	12,773 (48.3)	82 (39.2)	
female	13,813 (51.8)	13,694 (51.7)	121 (60.8)	
Race, (*n*/%)				0.111
Mexican American	3,882 (8.1)	3,864 (8.1)	18 (5.2)	
non-Hispanic black	5,618 (10.6)	5,565 (10.6)	53 (17.8)	
non-Hispanic white	12,252 (69.1)	12,149 (69.1)	103 (67.1)	
other Hispanic	2,341 (5.0)	2,325 (5.0)	16 (4.6)	
Other races	2,575 (7.2)	2,564 (7.2)	11 (5.3)	
Education, (*n*/%)				<0.001
Below high school	5,842 (14.0)	5,775 (14.0)	67 (21.3)	
High school	6,154 (23.2)	6,092 (23.1)	62 (43.4)	
Over high school	14,672 (62.8)	14,600 (62.9)	72 (35.3)	
Marital status, (*n*/%)				<0.001
Married/living with partner	16,228 (63.6)	16,130 (63.7)	98 (54.9)	
Never married	4,589 (18.1)	4,566 (18.1)	23 (9.0)	
Widowed/divorced/separated	5,851 (18.3)	5,771 (18.2)	80 (36.1)	
Hypertension, (*n*/%)	11,492 (37.8)	11,329 (37.6)	163 (74.5)	<0.001
Hyperlipidemia, (*n*/%)	18,781 (69.4)	18,610 (69.4)	171 (83.7)	<0.001
Energy intake kcal (median [IQR])	1974.50 [1535.29, 2536.47]	1975.00 [1536.00, 2538.00]	1731.71 [1213.19, 2161.41]	<0.001
Family income level, (*n*/%)				<0.001
<1.3	7,954 (20.7)	7,849 (20.6)	105 (39.5)	
>3.5	8,529 (44.1)	8,510 (44.2)	19 (11.5)	
1.3–3.5	10,185 (35.3)	10,108 (35.2)	77 (48.9)	
OBS, mean (SD)	20.99 (7.14)	21.02 (7.13)	17.13 (7.08)	<0.001
Dietary OBS. mean (SD)	16.73 (6.79)	16.75 (6.78)	13.54 (6.82)	<0.001
Lifestyle OBS, mean (SD)	4.26 (1.67)	4.27 (1.67)	3.58 (1.45)	<0.001

OBS, oxidative balance score.

### 3.2 Relationship between OBS and PSD

Weighted stepped logistic regression models presented in Table 3 revealed associations between quartiles of total, dietary, and lifestyle OBS and the risk of PSD. In the unadjusted model, quartiles of total, dietary, and lifestyle OBS were associated with a reduced risk of PSD. After multivariable adjustments, compared to the first quartile, the second quartile (OR: 0.46, 95% CI: 0.27–0.77) and the highest quartile (OR: 0.31, 95% CI: 0.15–0.67) of total OBS were significantly associated with a lower risk of PSD. Similarly, after adjustments, compared with the first quartile, the second quartile (OR: 0.45, 95% CI: 0.27–0.73) and the highest quartile (OR: 0.30, 95% CI: 0.15–0.61) of dietary OBS, as well as the highest quartile (OR: 0.28, 95% CI: 0.13–0.60) of lifestyle OBS, were significantly associated with a lower risk of PSD (all *p* for trend <0.05).

**Table 3:** Weighted logistics regression analysis of the association between OBS and PSD.

	Model 1	Model 2	Model 3
	OR (95% CI)	OR (95% CI)	OR (95% CI)
OBS			
Q1	Ref	Ref	Ref
Q2	0.38 (0.23, 0.62)	0.48 (0.30, 0.77)	0.46 (0.27, 0.77)
Q3	0.43 (0.26, 0.72)	0.63 (0.38, 1.04)	0.59 (0.33, 1.06)
Q4	0.20 (0.10, 0.39)	0.35 (0.17, 0.70)	0.31 (0.15, 0.67)
p for trend	<0.001	<0.001	0.002
Dietary OBS			
Q1	Ref	Ref	Ref
Q2	0.39 (0.25, 0.61)	0.48 (0.31, 0.75)	0.45 (0.27, 0.73)
Q3	0.50 (0.32, 0.79)	0.70 (0.44, 1.12)	0.63 (0.38, 1.06)
Q4	0.21 (0.12, 0.38)	0.36 (0.20, 0.66)	0.30 (0.15, 0.61)
*p* for trend	<0.001	<0.001	0.001
Lifestyle OBS			
Q1	Ref	Ref	Ref
Q2	0.83 (0.52, 1.35)	0.84 (0.53, 1.35)	0.89 (0.56, 1.43)
Q3	0.57 (0.25, 1.31)	0.60 (0.26, 1.39)	0.66 (0.28, 1.51)
Q4	0.18 (0.09, 0.39)	0.24 (0.11, 0.52)	0.28 (0.13, 0.6)
p for trend	<0.001	<0.001	0.002

Model 1 was a crude model without any adjustment; Model 2 partially adjusted for age, gender, race, marital status, and education level; Model 3 further adjusted for income level, energy intake, hyperlipidemia and hypertension based on Model 2.

### 3.3 Dose–response association between OBS and PSD risk

**Figure 2:**
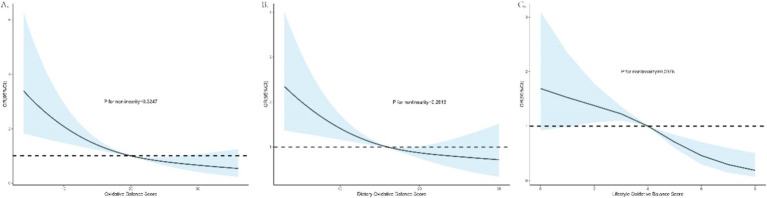
Dose–response associations of total, dietary, and lifestyle OBS with PSD risks. **(A)** OBS and PSD; **(B)** dietary OBS and PSD; **(C)** lifestyle OBS and PSD.

The dose–response relationships between total, dietary, and lifestyle OBS and PSD risk were evaluated using RCS analysis, employing weighted multivariable logistic regression adjusted for covariates, as depicted in Figure 2. The findings indicate a linear association between total, dietary, and lifestyle OBS and PSD risk, as evidenced by the non-significant *p*-values for nonlinearity (*p* > 0.05 for all). Higher OBS values were associated with lower odds ratios for PSD, suggesting that improvements in oxidative balance, through both diet and lifestyle, are linked to reduced PSD risk.

### 3.4 Sex-stratified subgroup analysis of the association between OBS and PSD odds

Table 4 presents the associations between OBS and PSD in male and female subgroups. After multivariable adjustments, total OBS, dietary OBS, and lifestyle OBS were inversely associated with the odds of PSD in females, whereas only lifestyle OBS was significantly inversely associated with the odds of PSD in males. In females, each unit increase in total, dietary, and lifestyle OBS was associated with a 6, 4, and 20% reduction in the odds of PSD, respectively. In males, each unit increase in lifestyle OBS was associated with a 28% reduction in the odds of PSD.

**Table 4:** Multivariable weighted logistics regression analysis of the association between OBS and PSD stratified by sex.

	OBS	Dietary OBS	Lifestyle OBS
	OR (95%CI)	OR (95%CI)	OR (95%CI)
Female	0.95 (0.92, 0.98)	0.96 (0.93, 0.99)	0.8 (0.7, 0.9)
Male	0.95 (0.91, 1.00)	0.98 (0.93, 1.03)	0.72 (0.61, 0.86)

Models were adjusted for age, gender, race, marital status, and education level, income level, energy intake, hyperlipidemia and hypertension.

## 4 Discussion

In this nationally representative population-based study, we identified a significant inverse association between higher OBS and the risk of PSD. Our findings demonstrated robust inverse associations between total, dietary, and lifestyle OBS and the risk of PSD. However, subtle variations were observed among different OBS components as well as across genders. Furthermore, dose–response analyses revealed linear relationships between total, dietary, and lifestyle OBS and PSD risk.

Chronic inflammation and oxidative stress are critical contributors to the pathogenesis of PSD (24). Stroke generates a significant amount of ROS, leading to oxidative stress in neural tissues, which causes lipid and protein peroxidation as well as DNA damage, believed to be a key mechanism underlying PSD development (25). The development of PSD is closely linked to oxidative stress and inflammatory mechanisms triggered by stroke. Mitochondrial dysfunction following a stroke further increases ROS production, damaging neurons and inducing apoptosis, ultimately impairing emotional regulation within the brain (26). Oxidative stress also activates the NLRP3 inflammasome, resulting in the release of pro-inflammatory cytokines such as IL-1β and IL-18, which exacerbate neuroinflammation and contribute to depressive-like behaviors (27). Additionally, elevated ROS disrupts redox balance, leading to sustained neuroinflammation that further damages neural networks involved in mood regulation (28). The systemic inflammation and oxidative stress triggered by ROS directly damages neural structures and inhibits synaptic plasticity and regeneration, ultimately increasing the risk of PSD (29). Dysregulation of the hypothalamic–pituitary–adrenal (HPA) axis, nuclear factor κB (NF-κB) signaling, and alterations in brain-derived neurotrophic factor (BDNF) expression further contribute to this process. Inflammation, driven by oxidative stress, is a key mechanism linking these factors to PSD (30). Metabolomics studies in animal models indicate that oxidative stress-related metabolites, such as docosahexaenoic acid (DHA), palmitic acid, and trimethylglycine, are associated with PSD (31). Moreover, the use of a corticotropin-releasing hormone receptor 1 (CRHR1) antagonist has been shown to reduce oxidative stress by inhibiting the Keap1-Nrf2-p62 pathway, thereby alleviating depression-like behaviors in PSD by reducing abnormal p62 accumulation (32). Animal model experiments have demonstrated that suppression of brain oxidative stress and inflammatory responses can mitigate the severity of PSD (33, 34).

Increasing evidence also highlights the role of oxidative stress and inflammation as interlinked processes that mutually amplify each other. Oxidative stress biomarkers (OBS) have emerged as important indicators of oxidative stress and inflammation in PSD. Systemic immunoinflammatory markers, such as the systemic immune-inflammatory index (SII), have been found to correlate significantly with PSD risk (35). Dietary and lifestyle strategies that reduce oxidative stress and inflammation, such as adherence to a high-quality diet rich in antioxidants, have been suggested to help prevent PSD and improve stroke prognosis (36, 37). Evidence indicates that unhealthy dietary and lifestyle habits frequently contribute to chronic inflammation, a critical factor in the pathogenesis of PSD (38–40). Antioxidant intake, such as vitamins B1, B2, niacin, B6, and B12, appears to have protective effects against PSD by mitigating oxidative stress and inflammation (41–44). Lifestyle factors also significantly impact PSD risk. Active physical activity (45), smoking cessation (46, 47), and maintaining a healthy weight are all associated with a reduced risk of PSD (48). Our findings indicate that lifestyle OBS is significantly inversely associated with PSD, emphasizing the importance of lifestyle modifications in preventing PSD.

Our study has several notable strengths, including the use of a nationally representative sample of the U.S. population and sex-subgroup analyses to enhance robustness. However, there are limitations: the cross-sectional design precludes causal inference, the self-reported nature of NHANES data introduces recall bias, and data on stroke severity and prescription medications were limited. Further research should explore the underlying mechanisms of PSD and the potential clinical applications of managing lifestyle and dietary factors to prevent PSD.

## 5 Conclusion

Data from this cross-sectional study indicates that OBS, which comprehensively reflects an individual’s overall oxidative stress burden, is inversely associated with the odds of PSD. Additionally, a sex difference was observed in these associations, with a greater protective effect noted for females. These findings underscore the importance of adhering to an antioxidant-rich diet and lifestyle for the prevention of PSD.

## Data Availability

Publicly available datasets were analyzed in this study. This data can be found here: centers for Disease Control and Prevention (CDC), National Center for Health Statistics (NCHS). Requests to access the datasets should be directed to https://wwwn.cdc.gov/nchs/nhanes/Default.aspx.

## Footnotes

^1^https://www.cdc.gov/nchs/nhanes/irba98.htm

^2^https://www.cdc.gov/nchs/nhanes/index.htm
